# Pyrimidine compounds BY4003 and BY4008 inhibit glioblastoma cells growth via modulating JAK3/STAT3 signaling pathway

**DOI:** 10.1016/j.neurot.2024.e00431

**Published:** 2024-08-16

**Authors:** Nisar Ahmad, Lixue Chen, Zixi Yuan, Xiaodong Ma, Xiaobo Yang, Yinan Wang, Yongshun Zhao, Huan Jin, Najib Khaidamah, Jinan Wang, Jiashuo Lu, Ziqi Liu, Moli Wu, Qian Wang, Yan Qi, Chong Wang, Yupu Zhao, Yang Piao, Rujie Huang, Yunpeng Diao, Sa Deng, Xiaohong Shu

**Affiliations:** a College of Pharmacy, Dalian Medical University, Dalian 116044, China; b College of Basic Medical Science, Dalian Medical University, Dalian 116044, China; c Institute of Integrative Medicine, Dalian Medical University, Dalian 116044, China; d Key Laboratories for Basic and Applied Research on Pharmacodynamic Substances of Traditional Chinese Medicine of Liaoning Province, Dalian Medical University, Dalian 116044, China; e The First Affiliated Hospital of Dalian Medical University, Dalian 116044, China

**Keywords:** Glioblastoma, Pyrimidine compounds, JAK3/STAT3 signaling, Anti-tumor

## Abstract

Glioblastoma (GBM) is a brain tumor characterized by its aggressive and invasive properties. It is found that STAT3 is abnormally activated in GBM, and inhibiting STAT3 signaling can effectively suppress tumor progression. In this study, novel pyrimidine compounds, BY4003 and BY4008, were synthesized to target the JAK3/STAT3 signaling pathway, and their therapeutic efficacy and mechanisms of action were evaluated and compared with Tofacitinib in U251, A172, LN428 and patient-derived glioblastoma cells. The ADP-Glo™ kinase assay was utilized to assessed the inhibitory effects of BY4003 and BY4008 on JAK3, a crucial member of the JAK family. The results showed that both compounds significantly inhibited JAK3 enzyme activity, with IC_50_ values in the nanomolar range. The antiproliferative effects of BY4003, BY4008, and Tofacitinib on GBM and patient-derived glioblastoma cells were evaluated by MTT and H&E assays. The impact of BY4003 and BY4008 on GBM cell migration and apoptosis induction was assessed through wound healing, transwell, and TUNEL assays. STAT3-regulated protein expression and relative mRNA levels were analyzed by western blotting, immunocytochemistry, immunofluorescence, and qRT-PCR. It was found that BY4003, BY4008 and Tofacitinib could inhibit U251, A172, LN428 and patient-derived glioblastoma cells growth and proliferation. Results showed decreased expression of STAT3-associated proteins, including p-STAT3, CyclinD1, and Bcl-2, and increased expression of Bax, a pro-apoptotic protein, as well as significant down-regulation of STAT3 and STAT3-related genes. These findings suggested that BY4003 and BY4008 could inhibit GBM growth by suppressing the JAK3/STAT3 signaling pathway, providing valuable insights into the therapeutic development of GBM.

## Graphical abstract

**Figure:**
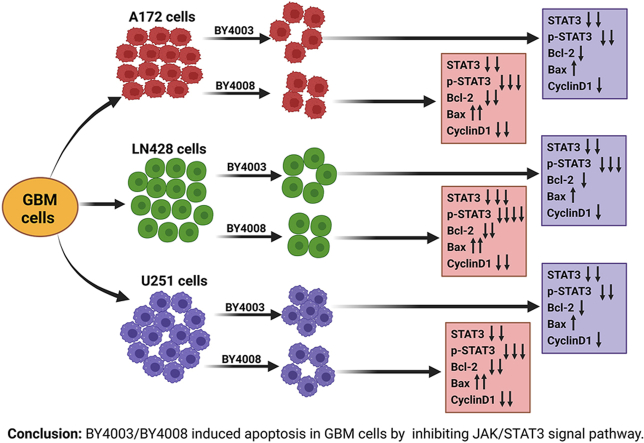

## Introduction

Glioblastoma is the most prevalent form of primary brain tumor in adults, representing 48.6% of all malignant brain tumors [1]. Due to its rapid, aggressive growth, incurability, and awful prognosis, the average lifespan of patients with GBM is estimated to be approximately 14–15 months [2]. The standard treatment for GBM involves surgery along with chemotherapy and radiotherapy [3]. However, the difficulty of removing invasive GBM tissues always results in its relapse after surgery [4]. Therefore, patients with GBM have to receive adjuvant chemotherapy after surgery to avoid tumor recurrence and extend their lives [5]. Although temozolomide (TMZ) is currently the most commonly used chemotherapeutic drug for glioblastoma, resistance to TMZ and severe side effects like infertility and bone marrow suppression are major problems with its therapy [6]. Therefore, it is urgently required to develop a novel, less toxic, and therapeutically effective strategy for the treatment of glioblastoma.

STAT3 is a key intracellular transcription factor belonging to the STAT family [7]. In the context of glioblastoma, STAT3 is crucial for tumor growth and progression [8]. Its abnormal activation has been linked with multiple human malignancies, including glioblastoma, breast, lung, and pancreatic cancer [9,10]. Normally, STAT3 is located in an inactive state within the cytoplasm [11]. However, when exposed to different stimuli like cytokines and growth factors [12], STAT3 undergoes activation by receptor-linked kinases such as JAK and non-receptor kinases like Src [13]. This activation results in STAT3 phosphorylation and the formation of homodimers [14]. These phosphorylated STAT3 dimers subsequently enter the nucleus and bind to DNA, activating target genes that regulate tumor cell proliferation, angiogenesis, metastasis, and anti-apoptotic responses [15]. STAT3 increases the transcription of genes involved in the regulation of cell proliferation and survival, which leads to uncontrolled tumor growth and drug resistance [16]. Therefore, the JAK/STAT3 pathway has been considered an ideal target for the treatment of glioblastoma [9,17]. Inhibiting STAT3 signaling can impede glioma cell proliferation, induce apoptosis, and enhance the sensitivity of tumor cells to conventional therapies [18]. Our previous research conducted on the STAT3 signaling pathway has provided evidence that inhibiting STAT3 signaling can induce apoptosis in glioma cells [19].

**Fig. 1:**
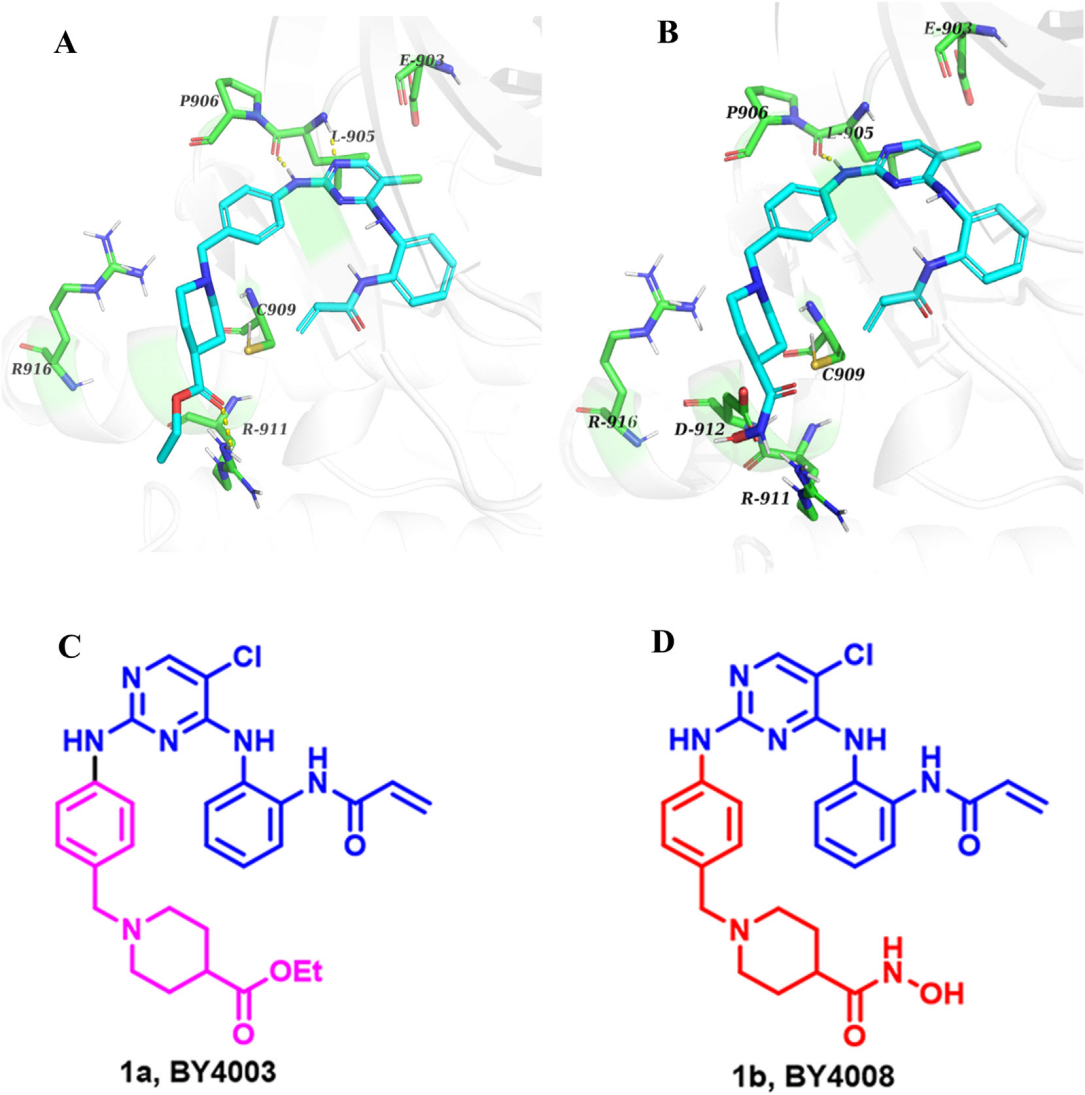
Putative binding interaction of BY4003 (A) and BY4008 (B) with JAK3. To determine how BY4003 and BY4008 bind to the catalytic core of JAK3, a molecular docking analysis was performed using AutoDock 4.2 software. The crystal structures of the prototype JAK3 molecule-binding protein (PDB code: 4Z16) served as a template for comparison. According to the docking studies, BY4003 and BY4008 interact significantly at the catalytic region of the kinase and may generate a covalent bond between Cys909 in JAK3 and the acrylamide (the area of the red circle in the figure). The 2-NH_2_ group in the pyrimidine core of the compounds formed strong hydrogen bonds with Leu905 in JAK3 (yellow dotted lines). The hydroxamic acid group in BY4008 formed a polar bond with the Asp912 position within the JAK3, resulting in a significant anti-JAK3 activity. (C) BY4003 chemical structure diagram (D) BY4008 chemical structure diagram.

Numerous small molecular JAK inhibitors, such as Tofacitinib, Ruxolitinib, and Pacritinib, are currently being studied in clinical trials [20]. JAK3 is the only member of the JAK family that has a cysteine residue (Cys909) at the Gatekeeper plus 7 (GK+7) position, which provides a novel approach for the investigation and development of selective covalent binding inhibitors [16]. The Cys909 position within the JAK3 protein represents a structurally significant target for covalent kinase inhibitors [21]. In our laboratory, a series of diphenyl-pyrimidine derivatives were designed and synthesized in previous studies, and most of them showed inhibitory activity on kinases like JAK3, FAK, EGFR, and BTK [[22], [23], [24]]. We made significant efforts to modify its structure and synthesized a variety of potent targets containing BY4003 and BY4008 compounds [25]. The molecular docking data showed that BY4003 and BY4008 exhibit a strong binding affinity for the kinase catalytic domain and are likely to form a covalent linkage between the acrylamide moiety and the cysteine residue at position 909 in JAK3 (Fig. 1A and B). It is expected that these compounds have an inhibitory effect on the JAK3/STAT3 signaling pathway. To investigate this inhibitory effect, we designed this project to evaluate the in-vitro effects of these two novel pyrimidine compounds on glioma cells. Furthermore, the purpose of this research is to determine whether this effect is regulated by the STAT3 signaling pathway.

## Materials and Methods

### Cell culture and reagents

Human glioblastoma cell lines A172, LN428, and U251 were obtained from the Cell Bank of the Chinese Academy of Sciences in Shanghai, China. The cells were grown in DMEM media with 1% penicillin-streptomycin and 10% FBS. The culture conditions were maintained at 37 ​°C in a humidified environment with 5% CO_2_. All chemicals and reagents used in the production of BY4003 and BY4008 were bought from Shanghai Wanghua Chemical Technology Co., Ltd. The Dulbecco's Modified Eagle Medium (DMEM) culture medium, trypsin (0.25%), and double antibiotic (penicillin/streptomycin) were obtained from the Hyclone Company in the United States. Fetal bovine serum was obtained from the American GIBCO Company; while PBS was acquired from Beijing Zhongshan Biotechnology Co., Ltd. Dimethyl sulfoxide (DMSO) was obtained from Sigma Corporation, USA. The MTT assay kit was procured from Key Gene Biotech. RIPA lysis buffer was obtained from Beyotime. The primary antibodies mentioned here were used: Bcl-2 (Proteintech), Bax (Proteintech), CyclinD1 (BIOSS antibodies), STAT3 (SAB Signal way antibody), p-STAT3 (SAB Signal way antibody), and GAPDH (Proteintech).

### Kinase enzymatic assay

The enzymatic analyses were conducted using the ADP-Glo™ assay kit with the JAK3 enzyme (Catalog. V3701) bought from Promega Corporation, USA. The assay was carried out following the manufacturer's protocol. Detailed protocols and active kinase data can be accessed at the following link:https://www.promega.com.cn/resources/protocols/product-information-sheets/n/JAK3kinaseenzyme system-protocol. The compounds used in the experiment were gradually diluted from the main stock solution of 1.0 ​μM in 100% DMSO, using a kinase reaction buffer. Concentrations ranging from 0.1 ​nM to 1000 ​nM were used. The IC_50_ analyses were carried out in triplicate. The experiment was conducted using a 384-well plate. To each well, 1 ​μL of the drug, 2 ​μL of mix 1 (containing ATP and Poly (Glu4, Tyr1) in a 1 ​× ​kinase buffer), and 2 ​μL of the kinase at a concentration of 0.05 ​ng/μL were added. The resulting mixture was incubated at 25 ​°C for 60 ​min. Subsequently, the reactions were stopped by adding 5 ​μL of mix 2 (ADP-Glo™ reagent) and incubating at 25 ​°C for 40 ​min. After that, 10 ​μL of the kinase detection reagent was added to each assay well and allowed to incubate for another 30 ​min at 25 ​°C. The luminescence was detected using a TriStar® LB942 Multimode Microplate Reader (BERTHOLD) with an integration time of 0.5–1s. Graph Pad Prism 5.0 was employed for curve fitting and data analysis.

### Cell proliferation assay

The MTT assay was used to figure out cell proliferation and cytotoxicity. Following the manufacturer's protocol, initially, the cells were grown in 96 well plates with 5 ​× ​10^3^ ​cells per well for 24 ​h. Following the adherence period, the cells were exposed to varying concentrations, ranging from 12.5 ​nM to 4 ​μM of BY4003 & BY4008 for 24, 48, and 72 ​h and with 1 ​nM–200 ​μM of Tofacitinib for 48 ​h. The plates were then incubated at 37 ​°C for 4 ​h, with 50 ​μL of MTT solution added to each well. The formazan crystals were dissolved with 150 ​μL of DMSO added to each well. The absorbance was recorded at 490 ​nm through a Multiskan GO spectrophotometer (Thermo, USA). The obtained values were presented as a percentage of cell growth inhibition % ​= ​[(1-OD of the treated groups/OD of the control groups) ​× ​100%].

### Hematoxylin and eosin (H&E) staining assay

The H&E assay was performed on glass coverslips containing cells. The cells were grown on glass coverslips inserted in a 6-well plate. After the incubation period, the culture medium containing BY4003 and BY4008 was added to the respective groups. Following 48 ​h of treatment, the glass coverslips were cleaned thrice with PBS and fixed with cold acetone. The coverslips were then kept at room temperature for 5 ​min and gently rinsed with PBS three times. Hematoxylin was added to stain the nuclei blue, followed by a water wash. The cells were immersed in the differentiation solution and subsequently stained with eosin dye after washing. The coverslips were then subsequently treated with 70% alcohol, 95% alcohol, anhydrous ethanol, ethanol-xylene, xylene (I), and xylene (II). Finally, the coverslips were sealed on glass slides with neutral gum, and morphological changes were observed under the microscope and photographed for documentation.

### Wound healing assay

To determine the effects of BY4003 and BY4008 on the migration of glioma cells, wound healing assays were performed. Cells were cultured in a six-well plate until they reached 70% confluence using standard growth media. The cell monolayer was then scratched with a sterile pipette tip, rinsed with PBS, and grown in DMEM medium with 2% FBS. Subsequently, cells were treated with specified concentrations of BY4003 and BY4008. The wounds were photographed at 0, 24, and 48 ​h after treatment. The alterations in the wound areas were examined and imaged using a light microscope (Nikon, Japan).

### Transwell assay

A transwell assay was performed to evaluate cell migration capability using Transwell chambers (Costar in New York, USA). Glioma cells A172, LN428, and U251 were seeded in the upper chambers with a density of 4 ​× ​10³ cells in 200 ​μL of serum-free medium with specific concentrations of BY4003 and BY4008. The lower chambers were filled with 600 ​μL of DMEM media supplemented with 10% FBS. After incubation at 37 ​°C for 24 and 48 ​h, the cells on the upper layer of the membrane were carefully scraped via swabs of cotton. The cells on the lower membrane were fixed with a 4% paraformaldehyde solution and then stained with a 0.5% crystal violet solution. The migrated cells were quantified and documented in five randomly chosen fields through a microscope.

### TUNEL assay

To investigate the impact of BY4003 and BY4008 on glioma cell apoptosis, cell-containing coverslips were rinsed three times with PBS before being permeabilized for 10 ​min with 0.1% Triton X-100. To identify apoptotic cells, a deoxynucleotidyl transferase dUTP-biotin nick end label assay (Beyotime Biotechnology, Shanghai, China) was utilized. TUNEL-labeled apoptotic cells were identified by red fluorescence. The cell photos were captured using a (Nikon ECLIPSE Ni–U) positive fluorescence microscope.

### Immunocytochemistry staining assay

An immunocytochemistry (ICC) examination was conducted on the glass coverslips collected from each experimental group using rabbit polyclonal antibodies against STAT3 (1:1000), p-STAT3 (1:500), CyclinD1 (1:500), Bcl-2 (1:500), and Bax (1:500). Briefly the coverslips were rinsed three times with PBS before permeabilized with 0.2% Triton X-100, for 10 ​min, then incubated with an appropriate amount of reagent-1 (endogenous peroxides blocker) for 10 ​min at 37 ​°C. Then the coverslips were kept overnight at 4 ​°C with appropriately diluted primary antibodies. The color reaction was carried out using DAB (3, 3’ diaminobenzidinetetra hydrochloride) and images were taken using a microscope (Nikon, ECLIPSE Ni–U). The staining results were evaluated based on the labeling intensity.

### Immunofluorescence staining assay

Initially, cells were cultured in six-well plates on glass coverslips and treated with BY4003 and BY4008 for 48 ​h. The coverslips were cleaned with PBS and fixed with cold acetone for 20 ​min after treatment. The cells were subsequently permeabilized by 0.1% Triton X-100 for 10 ​min before being treated with reagent-1 (an endogenous peroxidase blocker) for 10 ​min at 37 ​°C. The coverslips were then kept overnight with specific dilutions of primary antibodies, namely p-STAT3 (1:500), STAT3 (1:500), Bcl-2 (1:500), Bax (1:500), and CyclinD1 (1:400). The cells were then treated in the absence of light with suitable secondary antibodies (diluted 1:400) for 1 ​h. The nuclei were stained with DAPI for 5 ​min and the cells were observed using an immunofluorescence microscope and photographed.

### Western blot analysis

After a 48-h treatment with the specified concentrations of BY4003 & BY4008, the cells were subjected to lysis on ice for 30 ​min using a RIPA buffer that contained both protease and phosphatase inhibitors. The total protein content was calculated by the BCA. The protein was separated by 12% SDS-PAGE and transferred to PVDF membranes after being diluted with 5X loading buffer and boiled for 5 ​min. The membrane was blocked for 2 ​h in a 5% skim milk solution in TBS-T, then rinsed three times with TBS-T for 10 ​min before incubating overnight at 4 ​°C with primary antibodies. After washing with TBST, the membranes were soaked in secondary antibodies. Enhanced chemiluminescence detection reagents and a chemiluminescence imaging system (Tanon, Shanghai, China) were then used for detecting protein bands. The band's density was calculated by using Image J software.

### RNA isolation and qRT-PCR

According to the manufacturer's protocol, the total RNA was extracted from the cell after 48h treatment with BY4003 & BY4008 by using Trizol reagent (TaKaRa, Japan). Following the manufacturer's protocol (TaKara Bio, Kyoto, Japan) 2 ​μg of total RNA was reversely transcribed to cDNA in a 20 ​μL reverse transcription reaction mixture for qRT-PCR. Quantitative real-time PCR was performed according to the instructions provided with the SYBR Prime Script™kit (TaKaRa). All primers for specific genes used in this research were designed and synthesized by Takara Bio Inc. The primers' details are provided in Table 1. GAPDH was regarded as a reference gene. To determine the relative expression of target mRNA, the comparative threshold (Ct) method was employed, comparing the Ct values of the target mRNA with those of GAPDH (△Ct). The formula given below was used; △△CT ​= ​△CT sample －△CT calibrator. The gene's expression was mapped using a formula: 2^－△△CT^.

**Table 1:** Real-time PCR primers sequences.

Gene	Primer	Sequences
STAT3	Forward:	5′-TTCACTTGGGTGGAGAAGGACA-3′
	Reverse:	5′-CGGACTGGATCTGGGTCTTACC-3′
Bcl-2	Forward:	5′-CCCGTTGCTTTTCCTCTGG-3′
	Reverse:	5′-ATCCCACTCGTAGCCCCTCT-3′
Bax	Forward:	5′-CCCCGATTCATCTACCCTGC-3′
	Reverse:	5′-GAGCTAGGGTCAGAGGGTCA-3′
CyclinD1	Forward:	5′-TGTTCGTGGCCTCTAAGATGAA-3′
	Reverse:	5′-TCGGTGTAGATGCACAGCTTCT-3′
GAPDH	Forward:	5′-GCACCGTCAAGGCTGAGAAC-3′
	Reverse:	5′-TGGTGAAGACGCCAGTGGA-3′

### Patient-derived glioblastoma cell culture and treatments

Glioblastoma tumor tissues were collected from patients undergoing surgery at the First Affiliated Hospital of Dalian Medical University in China. Before tissue collection, all patients provided written informed consent, affirming their understanding of the study's purpose, procedures, potential risks, and benefits. The study protocol, including the collection and use of human tissue samples, was reviewed and approved by the Ethics Committee of the First Affiliated Hospital of Dalian Medical University (Approval No. PJ-KS-KY-2024-257). All experimental protocols were carried out strictly in accordance with this approval. The tissues were minced into small pieces and incubated in a solution containing collagenase IV (1 ​mg/mL) and DNase I (0.1 ​mg/mL) at 37 ​°C for 1–2 ​h. The digested tissue was filtered to get a single-cell suspension, which was then washed in PBS and centrifuged at 300 ​g for 5 ​min to obtain a cell pellet. The cell pellet was then dissolved in DMEM medium and seeded in a cell culture flask. The cells were maintained at 37 ​°C in a humidified atmosphere with 5% CO_2_ and changed every 3 days to remove non-adherent cells and debris. Once the cells reached 70–80% confluence, they were trypsinized and expanded the culture. To validate and characterize patient-derived glioblastoma cells, the expression of GFAP, a glioblastoma marker, was assessed by immunocytochemistry (ICC) and immunofluorescence techniques. To evaluate the impact of BY4003 and BY4008 on patient-derived glioblastoma cells and to compare it with the Tofacitinib the patient-derived glioblastoma cells were seeded overnight in 6-well plates on glass coverslips. The next day the patient-derived glioblastoma cells were treated with BY4003, BY4008, and Tofacitinib for 24 ​h. Following 24 ​h of treatment, the glass coverslips were cleaned thrice with PBS and fixed with cold acetone. The coverslips were then subsequently treated with H&E reagent to determine the growth inhibition and morphological alterations in patient-derived glioblastoma cells after treatment.

### Statistical analysis

The data obtained from this research was analyzed by Graph Pad Prism 7.0 software. The data was presented as a mean ​± ​SD. Student t-test was used for two sample comparisons. For multiple sample comparisons, one-way ANOVA was used. A *P*-value of <0.05 and ​<0.01 was regarded as statistically significant.

## Results

### BY4003 and BY4008 inhibit JAK3 activity and the proliferation of glioma cells

**Fig. 2:**
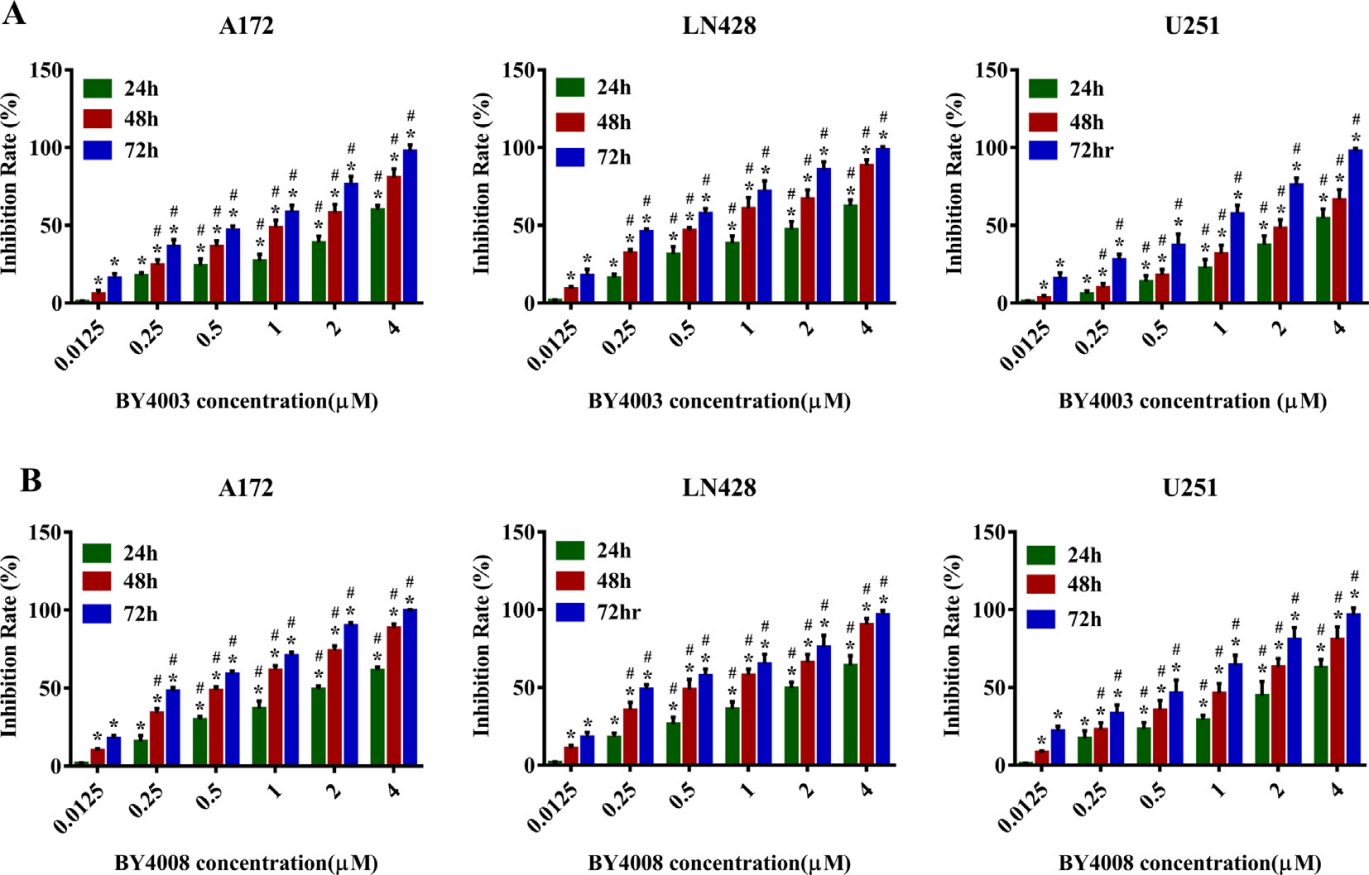
Inhibitory effects of BY4003 and BY4008 on glioma cell growth and proliferation. (A, B) illustrate the inhibitory impact of BY4003 and BY4008 on glioma cell growth and proliferation respectively. The obtained results were expressed as the mean ​± ​SD from three independent experiments. Statistical analysis showed significant variations, *∗, P* ​< ​0.05, compared with the former time point with the same BY4003 & BY4008 concentration; *#, P* ​< ​0.05, compared with the former BY4003 & BY4008 concentration at the same time point.

**Fig. 3:**
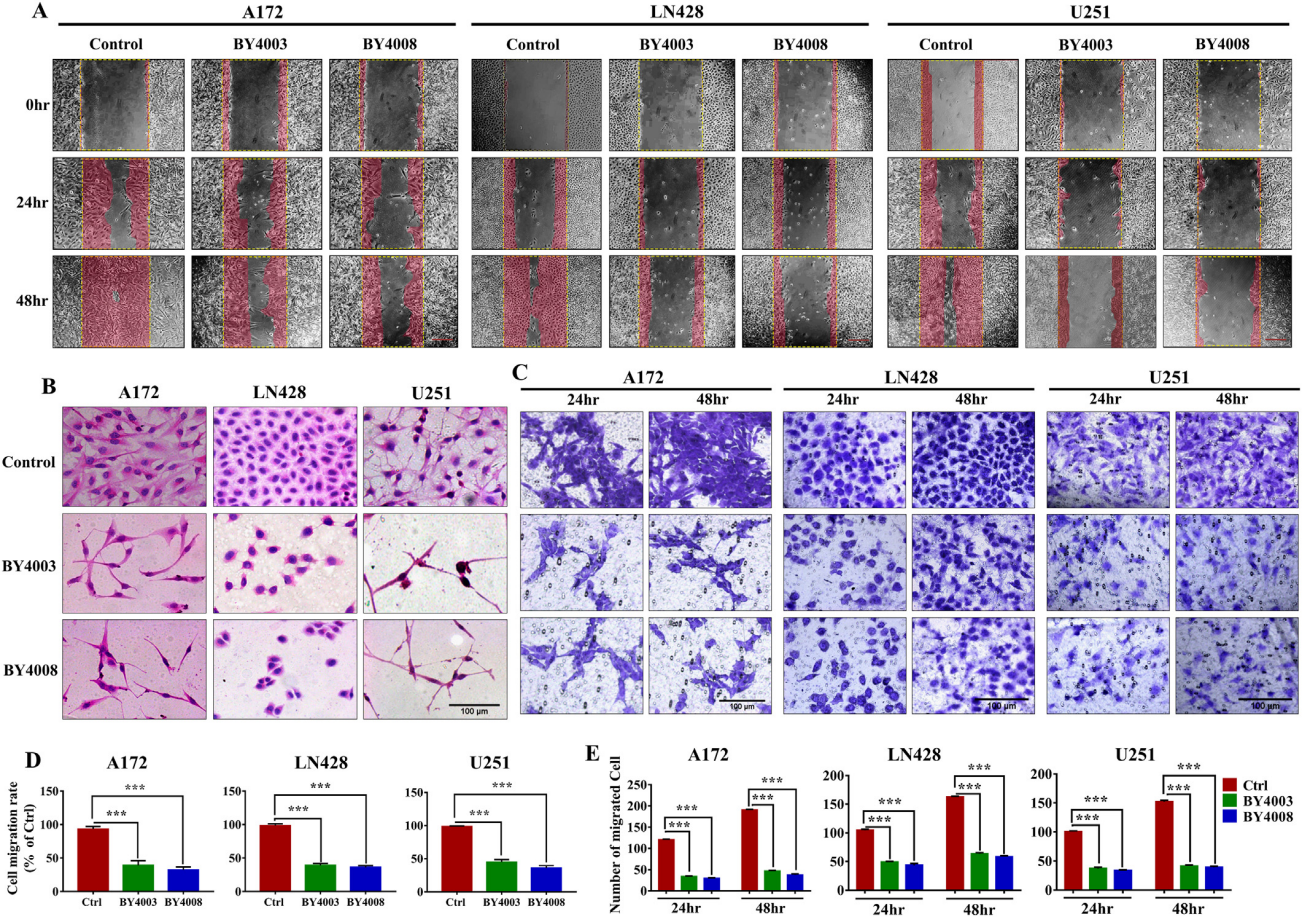
Inhibition of GBM cell migration BY4003 and BY4008. (A) illustrate the inhibitory effects of BY4003 and BY4008 on glioma cell migration, (D) represented its quantitative analysis by wound healing assay. (B) Showed morphological alteration in GBM cells treated BY4003 & BY4008. (C, E) indicated inhibitory effect and quantitative analysis of BY4003 and BY4008 on glioma cell migration by transwell assay. The results obtained were expressed as the mean ​± ​SD from three independent experiments. Data analysis revealed significant differences when comparing the treatment groups to the control group (∗∗∗, *p* ​< ​0.001). (Scale bar: 100 ​μm).

The inhibitory effects of BY4003 and BY4008 on JAK3 were assessed by ADP-Glo™ kinase assay. As expected, the enzymatic activity of JAK3 was significantly reduced by4003 and BY4008, with IC_50_ values of 16.32 ​nM and 26.20 ​nM, correspondingly. MTT assay was performed to analyze cell viability and evaluate the antiproliferative effects of BY4003, BY4008, and Tofacitinib on glioblastoma cells. MTT results showed that cell viabilities of U251, A172, and LN428 ​cells were decreased by4003 and BY4008 in a dose and time-dependent way (Fig. 2A and B). Additionally, MTT results showed that Tofacitinib significantly decreased the viabilities of GBM cells in a dose-dependent manner (Fig. S1). It was observed that the three human GBM cell lines showed different chemosensitivities to BY4003, BY4008, and Tofacitinib, with IC_50_ values for BY4008 were 2.42 ​μM, 1.98 ​μM, and 2.03 ​μM ​at 24 ​h; 1.02 ​μM, 0.55 ​μM, and 0.51 ​μM ​at 48 ​h; and 0.49 ​μM, 0.26 ​μM, and 0.25 ​μM ​at 72 ​h for U251, LN428, and A172, respectively. The IC_50_ of BY4003 for U251, LN428, and A172 were 3.6 ​μM, 2.02 ​μM, and 2.94 ​μM ​at 24 ​h; 2.11 ​μM, 0.58 ​μM, and 1.03 ​μM ​at 48 ​h; and 0.69 ​μM, 0.27 ​μM, and 0.50 ​μM ​at 72 ​h, respectively. In comparison, the IC_50_ values of Tofacitinib were 46.35 ​μM, 50.32 ​μM, and 44.99 ​μM for U251, LN428, and A172 at 48 ​h, respectively. In this study, Tofacitinib was used as a positive control to compare the antiproliferative effects of the pyrimidine compounds BY4003 and BY4008. Our results showed that BY4003 and BY4008 exhibited a stronger inhibitory effect than Tofacitinib on the three types of glioma cell lines (Table S1). Additionally, our results revealed that LN428 ​cells were more sensitive to BY4003 and BY4008 than U251 and A172 ​cells. Furthermore, growth inhibition and morphological alterations in U251, A172, and LN428 ​cells induced by BY4003 and BY4008 were examined via H&E staining. As shown in (Fig. 3B), after 48 ​h of incubation with BY4003 and BY4008, the glioma cells exhibited apparent cell abscission, increased cell spacing, cytoplasmic deformation, and fragmentation, along with significant morphological changes.

### BY4003 and BY4008 inhibit glioma cell migration

To further investigate the anti-tumor properties of BY4003 and BY4008, in-vitro wound healing and transwell studies were carried out to assessed their inhibitory impacts on glioma cell migration. Initially, wound healing experiments were carried out to assessed glioma cells' migratory abilities. Cell migration into the wound area was measured by the distance between wound edges before and after treatment with BY4003 and BY4008. The migration rates of cells exposed to BY4003 and BY4008 were compared to those in the control group and normalized to the migration rates of the Ctrl group. The results showed a notable decrease in GBM cell migration into the wound area in all three types of GBM cells treated with BY4003 and BY4008 (Fig. 3A–D). After 48 ​h of treatment, BY4003 decreased the migration of A172, LN428, and U251 ​cells to 39.19%, 39.20%, and 44.86%, respectively, while BY4008 decreased the migration rates to 32.15%, 36.16 %, and 35.81%, respectively (p ​< ​0.001, vs. Ctrl group) (Fig. 3A–D). Subsequently, the transwell experiment showed a significant reduction in the migration of glioblastoma cells after treatment with BY4003 and BY4008, as compared with the control group (Fig. 3C–E). After 48 ​h of incubation, the transwell assay revealed a marked reduction in the migration of cells treated with BY4003 and BY4008 compared to the control group. Specifically, the control group had 190 migrated A172 ​cells, whereas treatment with BY4003 and BY4008 reduced this number to 46 and 37 ​cells, respectively. Similarly, for LN428 ​cells, the control group exhibited 163 migrated cells, while the BY4003 and BY4008 treated groups showed a reduction to 63 and 58 migrated cells, respectively. For U251 ​cells, the number of migrated cells in the control group was 152, which decreased to 41 and 39 in the BY4003 and BY4008 treated groups, respectively (Fig. 3C–E). Both the wound healing and transwell assay results showed that BY4003 and BY4008 significantly inhibited glioblastoma (GBM) cell migration. The observed reduction in cell migration across A172, LN428, and U251 ​cell lines demonstrated the potential of BY4003 and BY4008 as effective anti-migratory agents for glioma treatment.

### BY4003 and BY4008 induce apoptosis in glioma cells

**Fig. 4:**
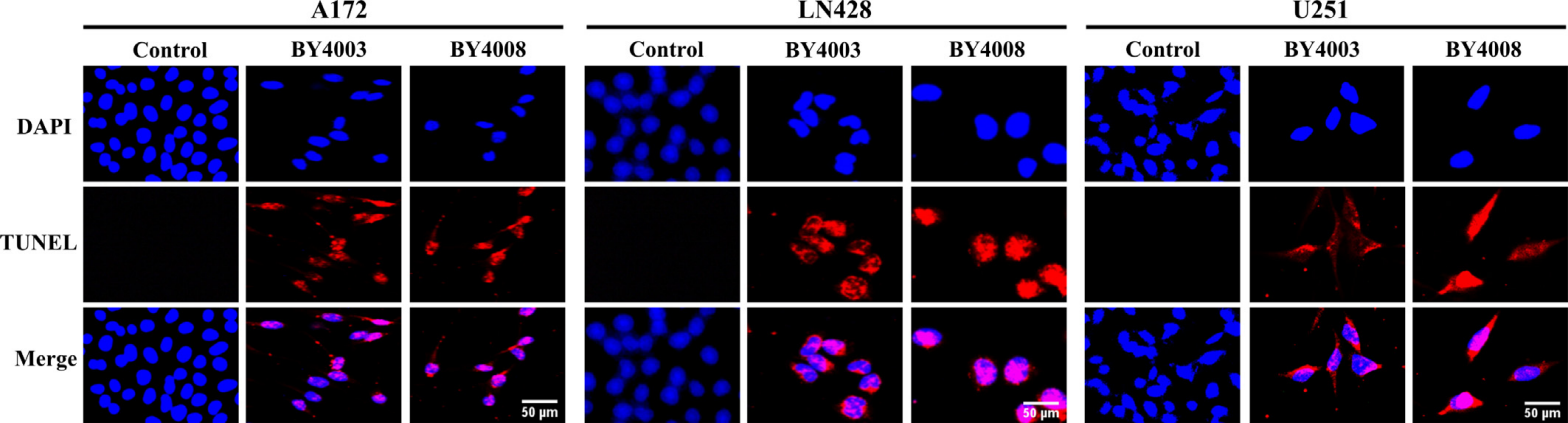
Induction of apoptosis in glioma cells treated with BY4003 and BY4008. TUNEL assay revealed a marked increase in apoptosis in GBM cells treated with BY4003 and BY4008 for 48 ​h (Scale bar: 50 ​μm).

Our results showed a substantial induction of apoptosis in the three types of glioma cells after being treated with BY4003 and BY4008 for 48h. The TUNEL assay revealed a higher number of apoptotic cells compared to the control group. As shown in (Fig. 4) the fluorescence signals observed in the BY4003 and BY4008 treated cells indicated DNA fragmentation, confirming the activation of apoptotic pathways. The rates of apoptosis were significantly higher in the BY4003 and BY4008 treatment groups compared to the control group, with BY4008 exhibiting a slightly stronger apoptotic effect than BY4003.

### Inhibitory effect of BY4003 and BY4008 on STAT3 signaling in glioblastoma

**Fig. 5:**
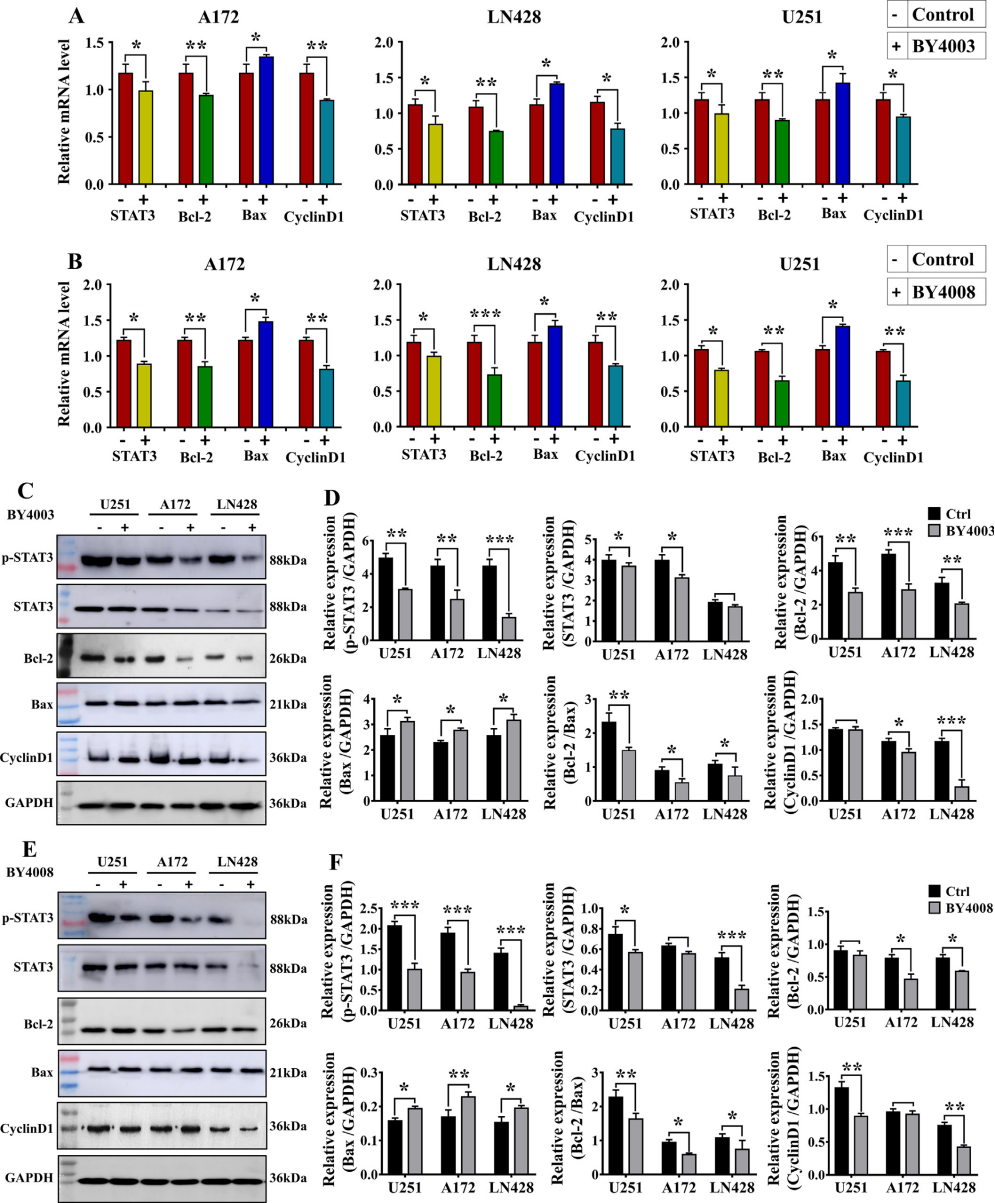
qRT-PCR and Western blot analysis of STAT3 signaling pathway-associated proteins in GBM cells. (A, B) showed the relative mRNA levels of STAT3, Bcl-2, Bax & CyclinD1 in A172, LN428, & U251 ​cells after 48h treatment with BY4003 and BY4008 respectively. (C, D) showed the expression levels of p-STAT3, STAT3, CyclinD1, Bcl-2, and Bax in U251, A172, and LN428 ​cells by western blotting and quantitative analysis after treatment with BY4003 for 48 ​h. (E, F) illustrated the effect of BY4008 on p-STAT3, STAT3, CyclinD1, Bcl-2, and Bax expression levels in U251, A172, and LN428 ​cells by western blotting and quantitative analysis. In the figure, (−) represents the Ctrl group, and (+) represents the treated group. GAPDH served as an internal control. All results were presented as the mean ​± ​standard deviation (SD) from three separate experiments. Statistical significance relative to the control group is denoted as follows: (∗*p* ​< ​0.05, ∗∗*p* ​< ​0.01, ∗∗∗*p* ​< ​0.001).

In GBM, uncontrolled activation of STAT3 has been observed which is related to glioblastoma growth, development, and a dismal prognosis. To determine the impact of BY4003 and BY4008 on STAT3 signaling, qRT-PCR was carried out on RNA isolated from glioma cells treated with BY4003 and BY4008 for 48 ​h. As represented in (Fig. 5A and B), the qRT-PCR results showed that after treatment with BY4003 and BY4008, the relative mRNA levels of STAT3, CyclinD1, and Bcl-2 were significantly decreased, while Bax relative mRNA levels significantly increased as compared to the control in A172, LN428, and U251 ​cells. These findings showed that BY4003 and BY4008 effectively inhibited the STAT3 signaling pathways.

To further validate the impact of BY4003 and BY4008 on the STAT3 signaling pathway, the expression levels of p-STAT3, STAT3, CyclinD1, Bcl-2, and Bax were determined by western blotting, immunocytochemistry, and immunofluorescence. The Western blot analysis revealed a significant reduction in the expression levels of p-STAT3 and STAT3 after treatment with BY4003 and BY4008, compared to the control group (Fig. 5C–F). Additionally, Western blot results also showed that the expression levels of CyclinD1 and Bcl-2 were downregulated, while Bax expression was upregulated in all three types of GBM cells after treatment (Fig. 5C–F). The Western blot results further confirmed and supported our hypothesis that BY4003 and BY4008 suppressed STAT3 activation and had an inhibitory effect on GBM cells.

**Fig. 6:**
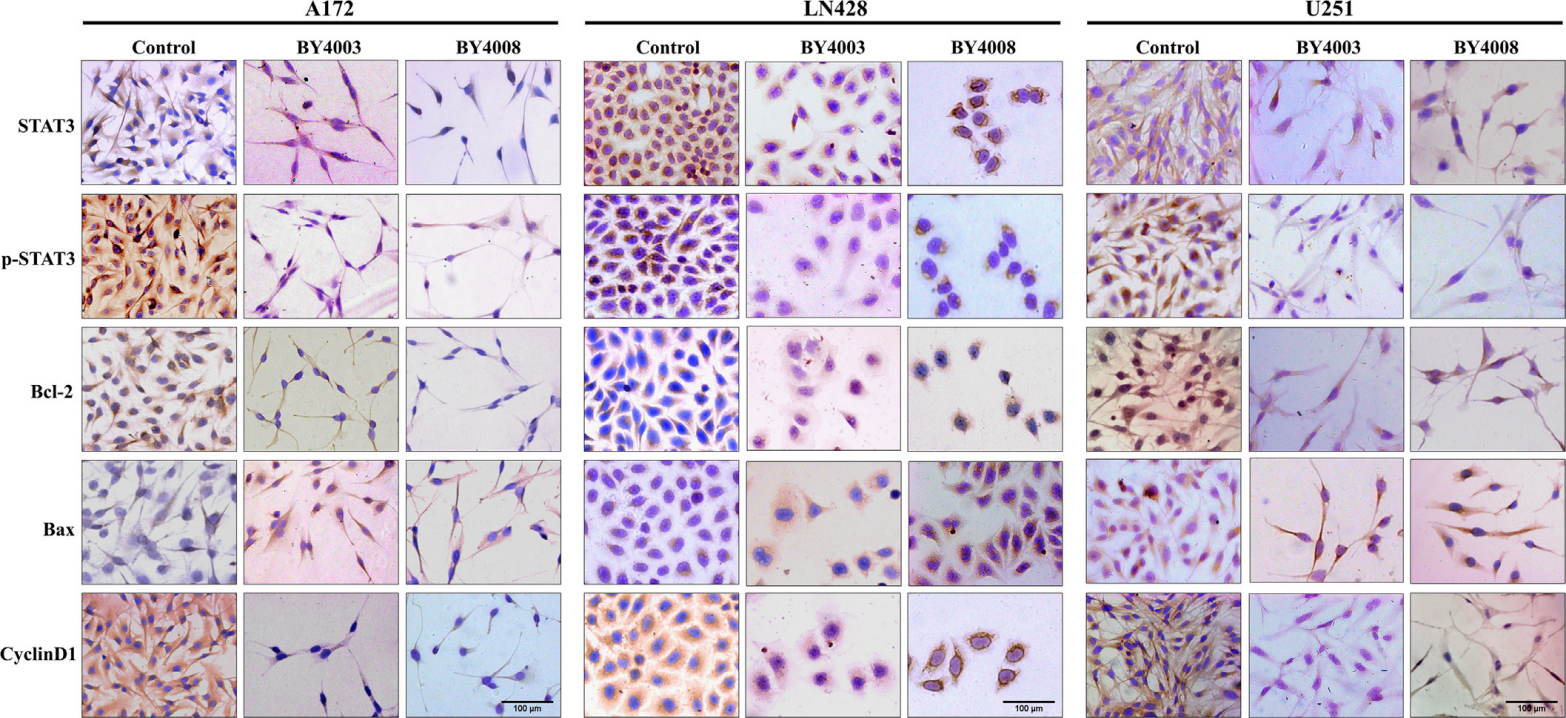
Immunocytochemical examination of STAT3 and its downstream proteins p-STAT3, Bcl-2/Bax, and CyclinD1 after 48h treatment with BY4003 and BY4008 in A172, LN428 & U251 ​cells. (Scale bar: 100 ​μm).

**Fig. 7:**
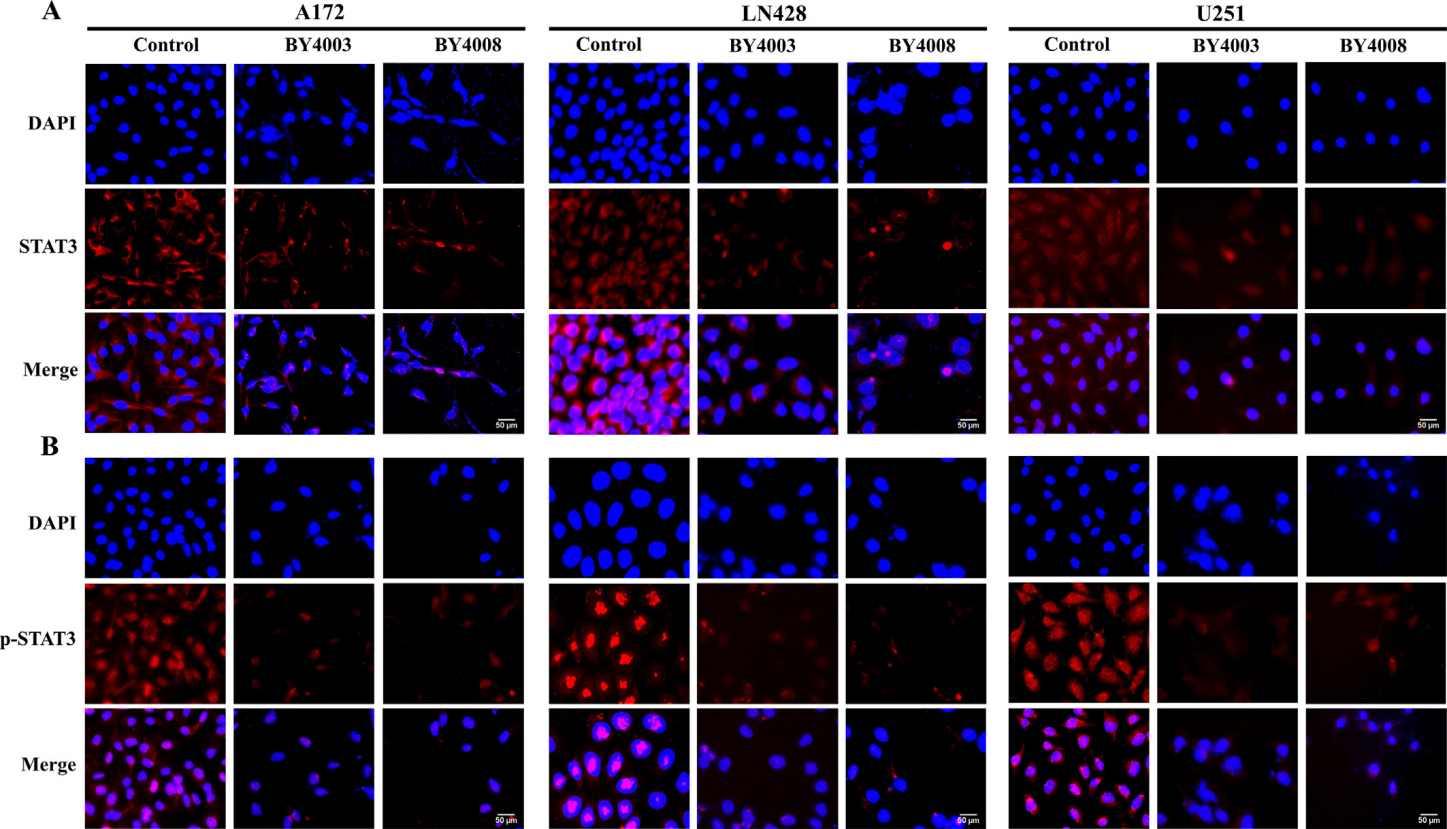
Immunofluorescence examination of STAT3 and p-STAT3 in A172, LN428 & U251 ​cells. (A, B) Showed considerable alterations in the expression levels of STAT3 and p-STAT3 treated with BY4003 & BY4008 for 48hr. (Scale bar: 50 ​μm).

In order to figure out the precise localization and comparative abundances of STAT3 and its signaling-associated downstream proteins, immunocytochemistry, and immunofluorescence staining assays were conducted on the same experimental group. The ICC results showed that p-STAT3 proteins were primarily localized around the nuclear envelope and within the nucleus in the control groups of A172, LN428, and U251 ​cells. However, after treatment with BY4003 and BY4008, p-STAT3 expression in the nucleus was significantly reduced. Additionally, ICC results also showed that under normal culture conditions, STAT3 was found both in the cytoplasm and nucleus of A172, LN428, and U251 ​cells, but its expression decreased after treatment with BY4003 and BY4008 (Fig. 6). Along with the ICC findings, the immunofluorescence results showed that in the control group, p-STAT3 was primarily localized around the nuclear membrane and within the nucleus of glioma cells. While its nuclear expression significantly decreased after treatment with BY4003 and BY4008 (Fig. 7B). Furthermore, immunofluorescence assay showed that STAT3 was typically expressed in the cytoplasm of A172, LN428, and U251 glioma cells, but its expression decreased after treatment with BY4003 and BY4008 (Fig. 7A).

**Fig. 8:**
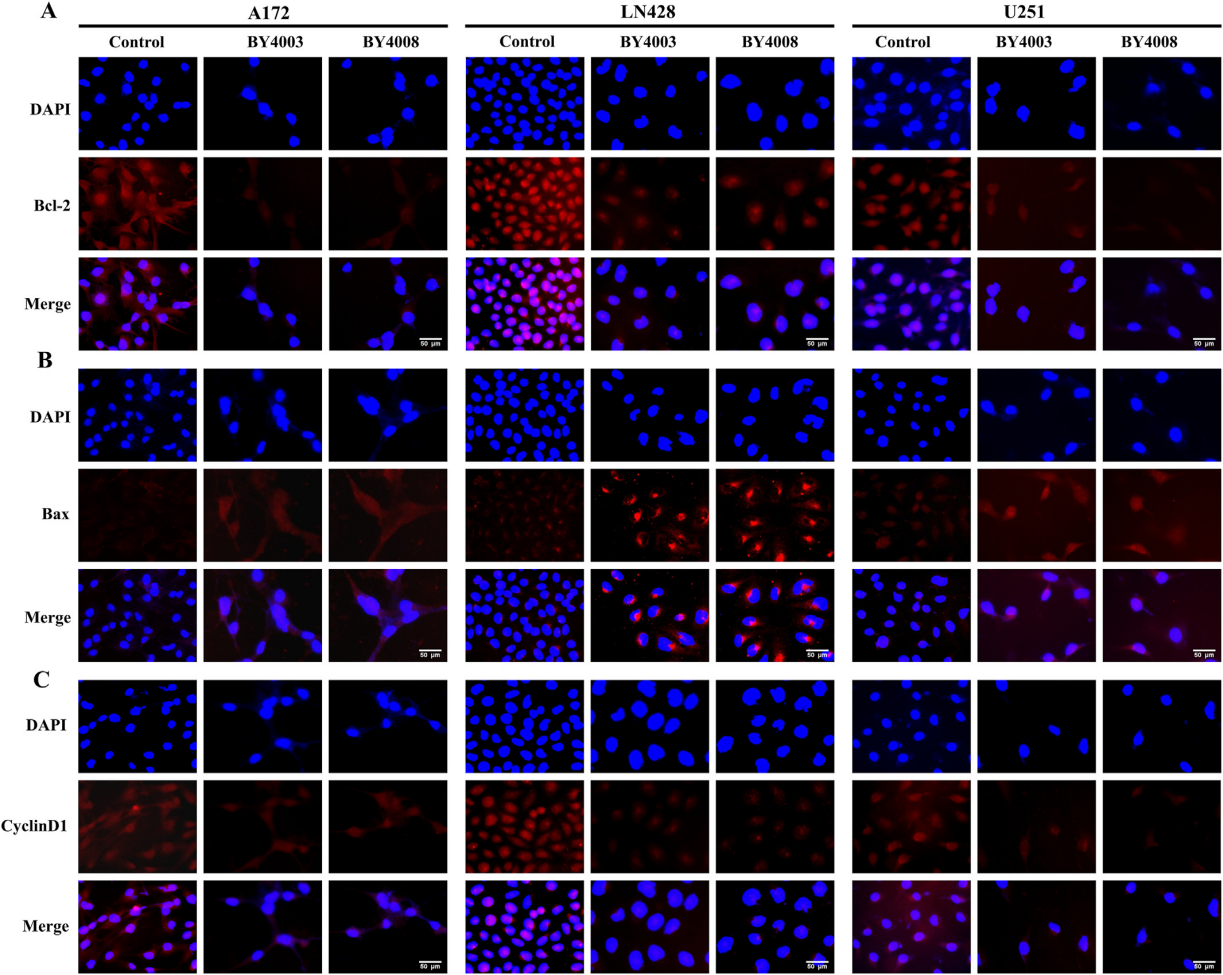
Immunofluorescence examination of Bcl-2, Bax, and CyclinD1 in A172, LN428 & U251 ​cells. (A, B, C) Showed the expression levels of Bcl-2, Bax, and CyclinD1 after 48h treatment with BY4003 and BY4008. (Scale bar: 50 ​μm).

To further study the impacts of BY4003 and BY4008 on STAT3 downstream proteins, the expression levels of CyclinD1, Bcl-2, and Bax were evaluated by immunocytochemistry (ICC) and immunofluorescence (IF) staining. The ICC results showed that CyclinD1 and Bcl-2 were normally expressed in the control groups, but their expression levels decreased after treatment with BY4003 and BY4008 (Fig. 6). Additionally, results also showed that pro-apoptotic protein Bax expression increased in the treated group relative to the control (Fig. 6). The results from immunofluorescence were consistent with those from immunocytochemistry. Immunofluorescence results further confirmed that CyclinD1 and Bcl-2 were normally expressed in the control groups, and their expression decreased after treatment with BY4003 and BY4008 (Fig. 8A–C). Furthermore, the results showed an increase in the expression of the pro-apoptotic protein Bax in the treated groups as compared to control groups (Fig. 8B), indicating that BY4003 and BY4008 induced apoptosis in glioma cells. The results obtained from immunocytochemical and immunofluorescence were consistent with Western blot results, which mutually confirmed that BY4003 and BY4008 had inhibitory effects on the STAT3 signaling pathway and induced apoptosis in the three types of GBM cells. Additionally, it was found that the decrease in p-STAT3 expression level in the BY4008 treated group was more apparent than BY4003 treated group, which showed that BY4008 had strong inhibitory effects on p-STAT3 as compared with BY4003 in GBM cells.

### Inhibitory effects of BY4003, BY4008, and tofacitinib on patient-derived glioblastoma cells

**Fig. 9:**
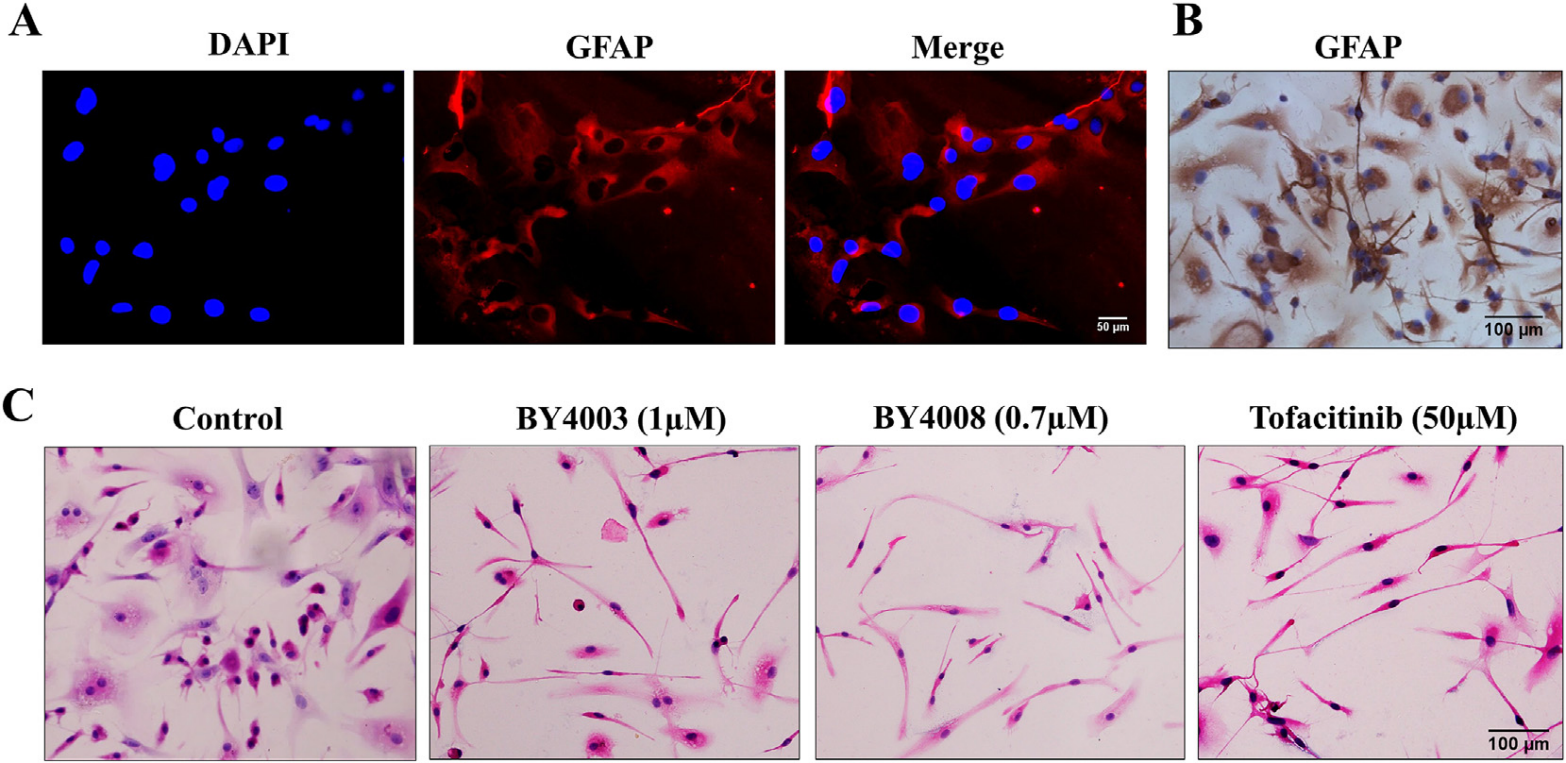
Inhibitory effects of BY4003, BY4008, and tofacitinib on patient-derived glioblastoma cells. Examination of GFAP expression by immunofluorescence (A) and immunocytochemistry (B) in patient-derived glioblastoma cells. Morphological alteration in patient-derived glioblastoma cells (C) treated with BY4003, BY4008, and Tofacitinib.

As shown in Fig. 9A & B, the ICC and IF results showed positive staining for GFAP confirming the glioblastoma phenotype of the patient-derived glioblastoma cultured cells. Additionally, the pathology report from the hospital also confirmed that the patient-derived glioblastoma cells retained the characteristic features of glioblastoma. This collective validation confirmed that the patient-derived glioblastoma cells retain characteristic features of glioblastoma, providing a reliable model for further research and therapeutic analysis.

As shown in Fig. 9C, after 48hr treatment with BY4003, BY4008, and Tofacitinib the patient-derived GB cells exhibited apparent cell abscission, increased intracellular spacing, cytoplasmic deformation, and fragmentation, along with significant morphological changes. These observations revealed that BY4003 and BY4008 significantly inhibited the growth and proliferation of patient-derived GBM cells. Furthermore, BY4003 and BY4008 showed higher potency than Tofacitinib, because they inhibit the growth and proliferation of patient-derived glioblastoma cells at very low concentrations (1 ​μM and 0.7 ​μM, respectively), as compared to Tofacitinib which required a significantly higher concentration of 50 ​μM to achieved the similar effects.

## Discussion

Glioblastoma multiform is an intracranial malignancy with an extremely poor prognosis [26]. Due to the difficulty of surgically removing highly aggressive GBM tissues, adjuvant chemotherapy has been extensively utilized to prevent tumor recurrence [27]. Surgery, radiation, and chemotherapy are currently recommended treatments for glioblastoma [28]. As a primary chemotherapeutic agent, temozolomide (TMZ) unfortunately failed to prevent the emergence of resistance and disastrous relapses [29,30]. Even with intensive therapeutic interventions including surgery, radiation, and chemotherapy, GBM patient survival had not significantly improved [31], and the majority of GBM patients underwent recurrence, which might be potentially fatal [32].

Research showed that STAT3 plays an important role in regulating the expression of genes associated with various types of cancer [33,34]. Compared to normal brain tissues, glioma tissue and cell lines had an elevated level of phosphorylated STAT3, which exacerbated glioma and contributed to drug resistance in malignant glioma cells [35,36]. STAT3-associated downstream proteins including CyclinD1, Bcl-2, and Bax could control multiple processes essential for the progression of cancer, such as tumor formation, growth, cell death, and differentiation [37]. Several studies showed that p-STAT3 is over-expressed in the majority of GBM patients and associated with mesenchymal differentiation and worse prognosis in gliomas [38], making the STAT3 signaling pathway an important therapeutic target for glioma treatment [39,40]. STAT3 increases the expression of genes that regulate cell proliferation and survival, leading to uncontrolled tumor growth and drug resistance [38]. Therefore, the JAK/STAT3 signaling cascade has been considered a prime therapeutic target for glioblastoma intervention. Studies showed that inhibiting JAK/STAT3 signaling can impede glioma cell proliferation, induce apoptosis, and enhance the sensitivity of tumor cells to conventional therapies. Clinical trials are currently going on to study numerous small molecular JAK inhibitors, including Tofacitinib, Ruxolitinib, and Pacritinib [41]. However, their clinical efficacy is constrained by several challenges, including limited blood-brain barrier penetration, off-target effects, resistance development, and immunosuppression [42]. To overcome these challenges, there is an urgent need to develop a safe and more effective novel therapeutic strategy that can improve patient survival and reduced GBM cell resistance. Aligned with this concept, two new pyrimidine BY4003 and BY4008 compounds were synthesized in our laboratory that showed a significant reduction in tumor progression. BY4008 had been shown to mediate its antitumor effects in colorectal cancer primarily by inhibiting STAT3 signaling [25]. Thus, the impacts of BY4003 & BY4008 on STAT3 signaling in GBM cells were studied.

To evaluate the impact of BY4003 and BY4008 on glioblastoma, the chemosensitivity of GBM cells to BY4003, and BY4008 was initially assessed. Our study revealed that treatment with BY4003 and BY4008 decreased the cell viability of U251, A172, and LN428 ​cells in a dose- and time-dependent manner (Fig. 3A and B). We observed that the three human GBM cell lines showed different chemosensitivities toward BY4003 and BY4008, with LN428 ​cells exhibiting greater sensitivity to both compounds compared to the A172 and U251 ​cell lines. Additionally, our findings showed that BY4008 exhibited a little strong anti-tumor effect on GBM cells compared to BY4003. Our study also revealed that BY4003 and BY4008 significantly suppress the growth, proliferation, and survival abilities of LN428 ​cells, which have shown resistance toward temozolomide and resveratrol, in our previous study [19]. Along with GBM cells, the results from patient-derived glioblastoma cells revealed that BY4003 and BY4008 significantly inhibited the growth and proliferation of patient-derived glioblastoma cells, with very lower concentrations than Tofacitinib indicated that BY4003 and BY4008 are the most potent, therapeutically sensitive and effective JAK3/STAT3 inhibitor having therapeutic potential to treat glioblastoma. In this study, Tofacitinib was used as a positive control, and its results are shown in the supplementary data.

Apoptosis is a vital physiological process of cells that plays a key role in maintaining cellular homeostasis by ensuring an important balance between apoptosis and proliferation [43]. This process not only removed harmful or superfluous cells but also significantly contributed to the prevention of cancer [44]. Dysregulation of apoptosis is a hallmark of cancer, resulting in uncontrolled cell proliferation and growth of malignant tumors [45]. Numerous medicines showed their anti-cancer properties by facilitating cellular apoptosis [46]. The results from our tunnel assay showed that BY4003 and BY4008 had the ability to induce apoptosis in A172, LN428, and U251 ​cells. Additionally, both compounds exhibited the ability to induce apoptosis in TMZ-resistant LN428 ​cells, indicating their potential as potent anticancer agents for GBM treatment.

One of the key causes of the increased mortality related to GBM is early metastasis [47]. Tumor metastasis is a complicated process, and cell migration is essential for the initial phase [48,49]. The results of our wound healing and transwell assays showed that BY4003 and BY4008 significantly inhibited the migration of A172, LN428, and U251 glioma cells. Subsequent investigations revealed that BY4003 and BY4008 also inhibited glioma cell proliferation, which is crucial in the progression of glioblastoma.

Our previous research has shown that the JAK-STAT3 signaling cascade is vital for glioblastoma tumorigenesis, proliferation, chemo-resistance, and immune cell infiltration [34]. On receiving the upstream signal stimulation, JAK is activated by tyrosine phosphorylation, which additionally causes STAT3 phosphorylation [50]. Phosphorylated STAT3 then formed a dimer and entered the nucleus to regulate a variety of target genes involved in cancer growth [51]. Our results from immunocytochemistry, immunofluorescence, and western blotting analysis showed that treating LN428, A172, and U251 ​cells with BY4003 and BY4008 inhibited the STAT3 activation and decreased the STAT3, p-STAT3, Bcl-2, and CyclinD1 expression levels while increased the expression of Bax. After treatment with BY4003 and BY4008, the Bcl-2/Bax ratio significantly decreased, revealing that the pyrimidine compounds BY4003 and BY4008 induced apoptosis in GBM cells. In addition to immunofluorescence, immunocytochemistry, and Western blot analyses, qRT-PCR findings also revealed that treatment with BY4003 and BY4008 significantly decreased the relative mRNA levels of STAT3, CyclinD1, and Bcl-2, while increasing the relative mRNA levels of Bax. The qRT-PCR results further confirmed and justified our findings from ICC, immunofluorescence, and Western blot, providing strong evidence that BY4003 and BY4008 effectively suppressed STAT3 activation and inhibited GBM cell growth and proliferation. This validation confirmed that STAT3 was a therapeutic target for BY4003 and BY4008 in glioblastoma.

**Fig. 10:**
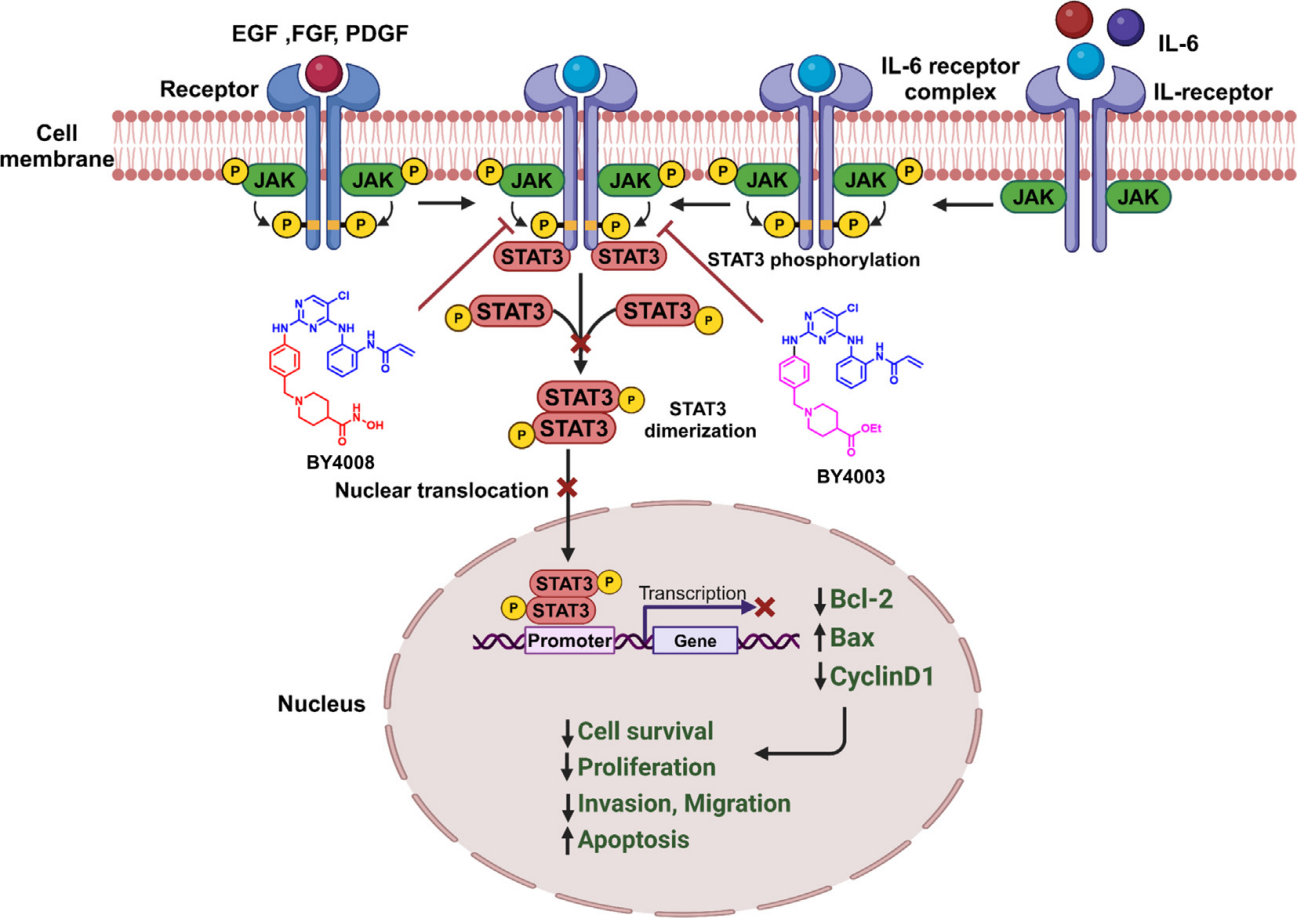
A schematic figure illustrates STAT3 signaling in glioblastoma and the mechanisms of action of BY4003 and BY4008 in glioblastoma. STAT3 signaling is initiated by the binding of different ligands, such as IL-6 and epidermal growth factor, to their cell surface receptors, resulting in the STAT3 phosphorylation. The phosphorylated STAT3 forms homodimers, translocated to the nucleus, binds to DNA, and modulates the expression of CyclinD1, Bcl-2, and Bax genes that are involved in tumor cell proliferation, survival, migration, drug resistance, and anti-apoptotic responses. BY4003 and BY4008 inhibited STAT3 phosphorylation, which leads to inhibition of glioma cell growth and proliferation.

In summary, our study revealed that BY4003 and BY4008 had a strong anti-tumor effect on A172, LN428, U251, and patient-derived glioblastoma cells by suppressing glioma cell migration, proliferation, and invasion. Additionally, our study found that BY4003 and BY4008 induced apoptosis and suppressed JAK3/STAT3 signaling activation, which could be the possible mechanism of action for these two newly synthesized pyrimidine compounds, as the activation of this pathway played a significant role in GBM progression (Fig. 10). Thus, our research introduced a novel therapeutic agent for the treatment of glioblastoma. As, our current study primarily focused on in-vitro cell line models, which are valuable for initial screening and mechanistic studies but do not fully replicate the complexity of in-vivo tumor environments. Therefore, additional research is needed to further evaluate its anti-tumor effect in in-vivo models and to explore the pharmacokinetics, biodistribution, and therapeutic potential of these compounds in animal models and clinical practice.

## Funding

This work was supported by the National Natural Science Foundation of China (81672945), the Education Department Foundation of Liaoning Province (LJKZ0827 and JYTQN2023153), and Dalian Medical University Interdisciplinary Research Cooperation Project Team Funding (JCHZ2023011).

## Institutional review board statement

Not applicable.

## Informed consent statement

Not applicable.

## Data availability statement

The datasets used and/or analyzed during the current study are available from the corresponding author on reasonable request.

## Author contributions

Conceptualization, X.S.; Methodology, N.A., L.C., and Y.W.; Software, N.A., H.J., N.K. and J.W.; Validation, X.M., M.W., Q.W. and C.W.; Formal analysis, N.A., J.L. and Y.Q.; Investigation, N.A., Y.Z., M.W., Q.W. and Y.P.; Resources, X.S. Y.Z. and L.C.; Data curation, R.H., Y.D. and S.D.; Writing—original draft preparation, N.A., L.C.; Writing—review and editing, X.S. and X.Y.; Visualization, N.A., Z.L. and N.K.; Supervision, X.S., L.C. and X.Y.; Project administration, X.S. and L.C.; Funding acquisition, X.S. and C.W. All authors have read and agreed to the published version of the manuscript.

## Declaration of competing interest

The authors declare that they have no known competing financial interests or personal relationships that could have appeared to influence the work reported in this paper.

## Figures and Tables

**Figure:**
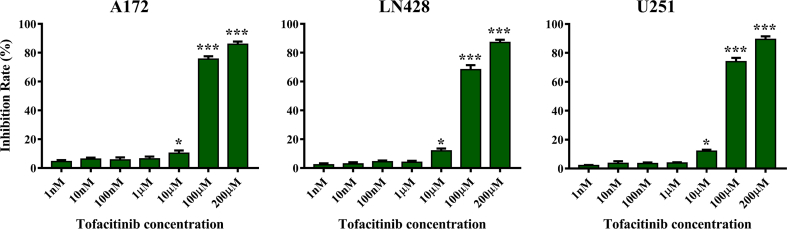
Fig. S1Inhibitory effects of Tofacitinib on glioma cell growth and proliferation.
