# Supplementary material for: Pyrimidine compounds BY4003 and BY4008 inhibit glioblastoma cells growth via modulating JAK3/STAT3 signaling pathway
Source: Neurotherapeutics. 2024 Aug 16;21(5):e00431. doi: 10.1016/j.neurot.2024.e00431 (PMC11579875; doi:10.1016/j.neurot.2024.e00431)
Supplement: Multimedia component 1 [file mmc1.docx]

**1. General procedure for the synthesis of BY4003 (1a) and BY4008 (1b)**

3-chloropropannoyl chloride (100 mmol) was gradually added to a mixture of potassium carbonate (50 mmol) and **2** (50 mmol) in acetonitrile under ice bath. After addition, it was allowed to stir for 60min continuously after warming to room temperature. The mixture was diluted with brine and ethyl acetate; the separated ethyl acetate layer was washed with brine and dried over anhydrous Na_2_SO_4_. After removing ethyl acetate under vacuum, the obtained organic substance was dispersed with petroleum ether to give intermediate **3**. **3** (40 mmol), Pd‒C (5% w/w) were added to methanol, catalytic hydrogenation was carried out at room temperature for overnight. Pd‒C was filtered out and the organic phase was removed under vacuum to get **4**. **4** (30 mmol), DIPEA (60 mmol) and 2, 4-dichloro-5-fluoropyrimidine/2, 4, 5-trichloropyrimidine (30 mmol) in acetonitrile, it was heated to 80^o^C gradually and stirred for 10 h. The precipitate was filtrated out and washed with water, and the obtained crude **5** could directly be used without further purification. Finally, a mixture of **5** (25 mmol), MeCN and TEA (200 mmol) was stirred under the condition of reflux for 6 h. Then water was gradually added to the cooled mixture and stirred for 30 min to get target **6**. A mixture of **6** (3.0 mmol), **7** or **8** (1.5 mmol), and *p*-TsOH (4.5 mmol) in EtOH was gradually heated to 70^o^C and stirred for 6 or 3 h. After reaction, the solvent was vacuum distilled and the residue was dispersed with saturated sodium bicarbonate/ethyl acetate. The organic phase was removed under vacuum to give the crude which was purified by (Prep-HPLC) to get the **1a** and **1b**.

The synthetic pathway for the compounds BY4003 and BY4008 is illustrated in (Figure 1). In brief, 2-nitroaniline (2) was conjugated to 3-chloropropionyl chloride through a trivial acyl substitution reaction to produce intermediate 3. The nitro arene group in compound 3 was reduced to the equivalent aniline by H_2_ in the presence of 10% Pd/C to produce compound 4, which then undergo a condensation reaction with 2,4,5-trichloropyrimidine to produce intermediate 5. Finally, the, elimination was performed to generate the main intermediate 6 bearing the acrylamide pharmacophore. In the presence of CH_3_ONa, compound 8 was synthesized via nucleophilic substitution with hydroxylamine hydrochloride and hydroxylamine hydrochloride. Finally, the final BY4003 (1a) or BY4008 (1b) was synthesized by nucleophilic aromatic substitution of the aryl chloride components of 6 with the aniline group of 7 or 8 catalyzed by p-TsOH. The structures of BY4003 (1a) and BY4008 (1b) were determined by ^1^H NMR, ^13^C NMR and HRMS spectrum's.

**Figure 1.** The synthetic route of BY4003 (1a) and BY4008 (1b). Reagents and conditions: (a) 3-chloropropionyl chloride, K_2_CO_3_ in CH_3_CN, from ice-bath to room temperature (r.t.), 95.0%; (b) 10%/Pd-C in MeOH, r.t.,10 h, 98.8%; (c) 2,4,5-trichloropyrimidine, DIPEA in CH_3_CN, 80 ^o^C for 10 h, 92.4%; (d) TEA in CH_3_CN, 80 ^o^C for 10 h, 96.1%; (e) Pd-C/10% in MeOH, r.t., 10 h, 98.0%; (f) NH_2_OH HCl, CH_3_ONa in MeOH, from ice-bath to r.t. for 2~3 h, ~100%; (g) TsOH in EtOH, 70 ^o^C for 3 h, 11%; (h) TsOH in EtOH, 70 ^o^C for 3 h, 56.3%.

***ethyl 1-(4-((4-((2-acrylamidophenyl) amino)-5-chloropyrimidin-2-yl)amino)benzyl)piperidine-4-carboxylate (1a)***

Yield 56.3%; off-white solid.^1^H NMR(400 MHz, DMSO‒*d*_6_): *δ*10.21(s, 1H), 9.28(s, 1H), 8.52(s, 1H), 8.12(s, 1H), 7.74(dd, *J*=7.98, 1.21 Hz, 1H),7.47(dd, *J*=7.98, 1.21 Hz, 1H),7.46(d, *J*=8.42 Hz, 2H),7.32(td, *J*=7.51, 1.40 Hz, 1H),7.27(td, *J*=7.51, 1.40 Hz, 1H),7.00(d, *J*=8.42 Hz, 2H),6.51(dd, *J*=17.02, 10.21 Hz, 1H),6.32(dd, *J*=17.02, 1.74 Hz, 1H), 5.80(dd, *J*=10.21, 1.74 Hz, 1H), 4.05(q, *J*=7.08 Hz, 2H),3.31(s, 2H), 2.70(d, *J*=10.74 Hz, 2H),2.26(tt, *J*=11.06, 3.84 Hz, 1H),1.92(t, *J*=10.80 Hz, 2H), 1.77(d, *J*=12.96 Hz, 2H), 1.53(qd, *J*=11.22, 3.42 Hz, 2H),1.19(t, *J*=7.08 Hz, 3H);^13^C NMR(100 MHz, DMSO‒*d*_6_): *δ*174.90, 164.70, 158.19, 156.56, 154.95, 132.03, 131.95, 131.45, 129.23(2C), 128.21, 127.98, 125.96(2C), 124.89, 119.08(2C), 104.41,62.38, 60.24, 52.55(2C), 40.81, 28.46(2C), 14.57.HRMS (ESI^+^) for C_28_H_31_ClN_6_O_3_, [M+H]^+^calcd: 535.2219, found: 535.2220.

***1-(4-((4-((2-acrylamidophenyl)amino)-5-chloropyrimidin-2-yl)amino)benzyl)-N-hydroxypiperidine-4-carboxamide (1b)***

Yield 11.0%; off-white solid.^1^H NMR(400 MHz, DMSO‒*d*_6_): *δ*10.38(s, 1H), 10.20(s, 1H), 9.27(s, 1H), 8.68(s, 1H), 8.52(s, 1H), 8.12(s, 1H), 7.76(d, *J*=7.60 Hz, 1H), 7.48(d, *J*=8.16 Hz, 3H), 7.32(t, *J*=7.20 Hz, 1H), 7.28(t, *J*=7.56 Hz, 1H), 7.03(d, *J*=8.28 Hz, 2H), 6.52(dd, *J*=16.92, 10.12 Hz, 1H), 6.34(dd, *J*=16.92, 1.36 Hz, 1H), 5.82(dd, *J*=10.12, 1.36 Hz, 1H), 3.33(s, 2H), 2.80(d, *J*=10.76 Hz, 2H), 1.98‒1.93(m, 1H), 1.86‒1.82(m, 2H), 1.63‒1.56(m, 4H); ^13^C NMR(100 MHz, DMSO‒*d*_6_): *δ* 172.04, 164.74, 158.22, 156.58, 154.95, 139.61, 132.50, 131.95, 131.47, 131.37, 129.25(2C), 128.20, 127.96, 125.97(2C), 124.94, 119.14(2C), 104.45, 62.43, 52.97(2C), 40.00, 28.89(2C).


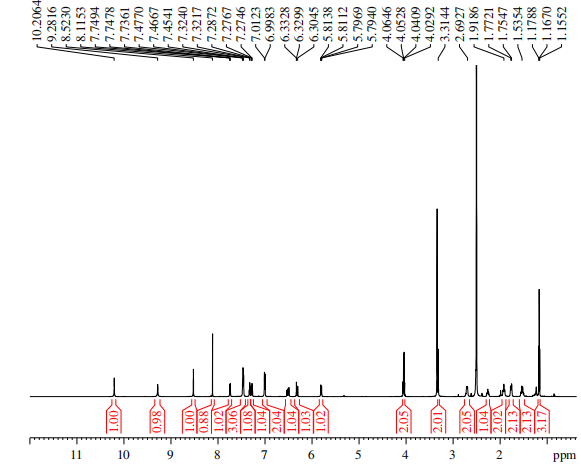


**Figure**
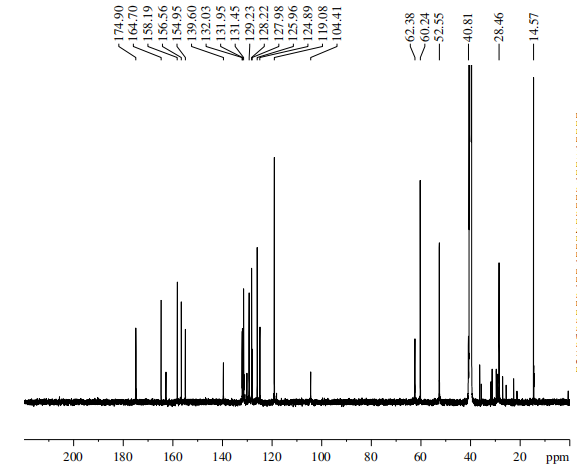
**2.** The ^1^H NMR spectrum of **1a**

**Figure 3.** The ^13^C NMR spectrum of **1a**

**
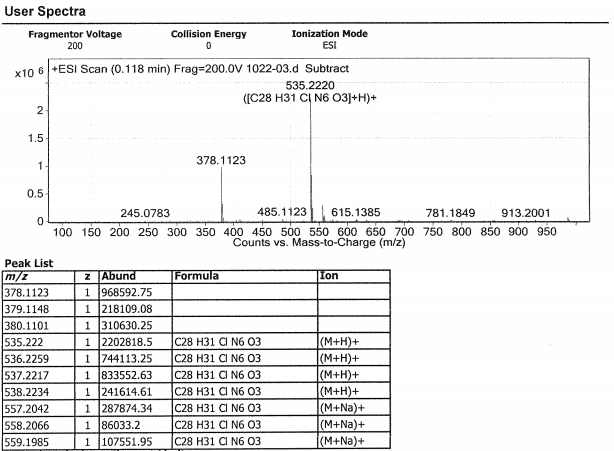
**

**Figure**
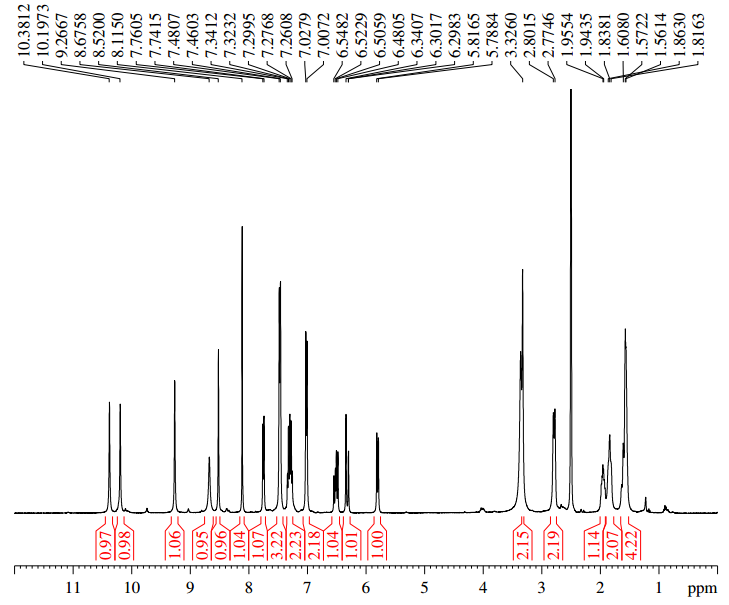
**4** The HR MS of **1a**

**Figure 5.** The ^1^H NMR spectrum of **1b**

**
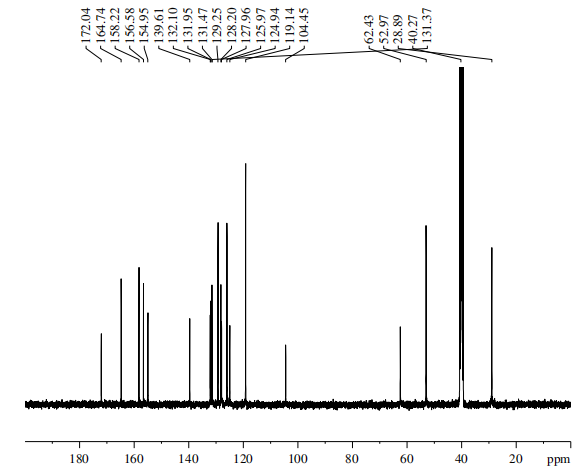
**

The ^13^C NMR spectrum of **1b**

**Figure 6.** The ^13^C NMR spectrum of **1b**
