# Supplementary material for: Pyrimidine compounds BY4003 and BY4008 inhibit glioblastoma cells growth via modulating JAK3/STAT3 signaling pathway
Source: Neurotherapeutics. 2024 Aug 16;21(5):e00431. doi: 10.1016/j.neurot.2024.e00431 (PMC11579875; doi:10.1016/j.neurot.2024.e00431)
Supplement: Multimedia component 2 [file mmc2.docx]

**Table S1. IC_50_ values of BY4003, BY4008 and Tofacitinib in 48h-treatment glioblastoma cells**

| **Type of cells** | **BY4003** | **BY4008** | **Tofacitinib** |
| --- | --- | --- | --- |
| U251 | 2.11 µM | 1.02 µM | 46.35 µM |
| LN428 | 0.58 µM | 0.55 µM | 50.32 µM |
| A172 | 1.03 µM | 0.51 µM | 44.99 µM |
