# Supplementary figures and images for: Pyrimidine compounds BY4003 and BY4008 inhibit glioblastoma cells growth via modulating JAK3/STAT3 signaling pathway
Source: Neurotherapeutics. 2024 Aug 16;21(5):e00431. doi: 10.1016/j.neurot.2024.e00431 (PMC11579875; doi:10.1016/j.neurot.2024.e00431)

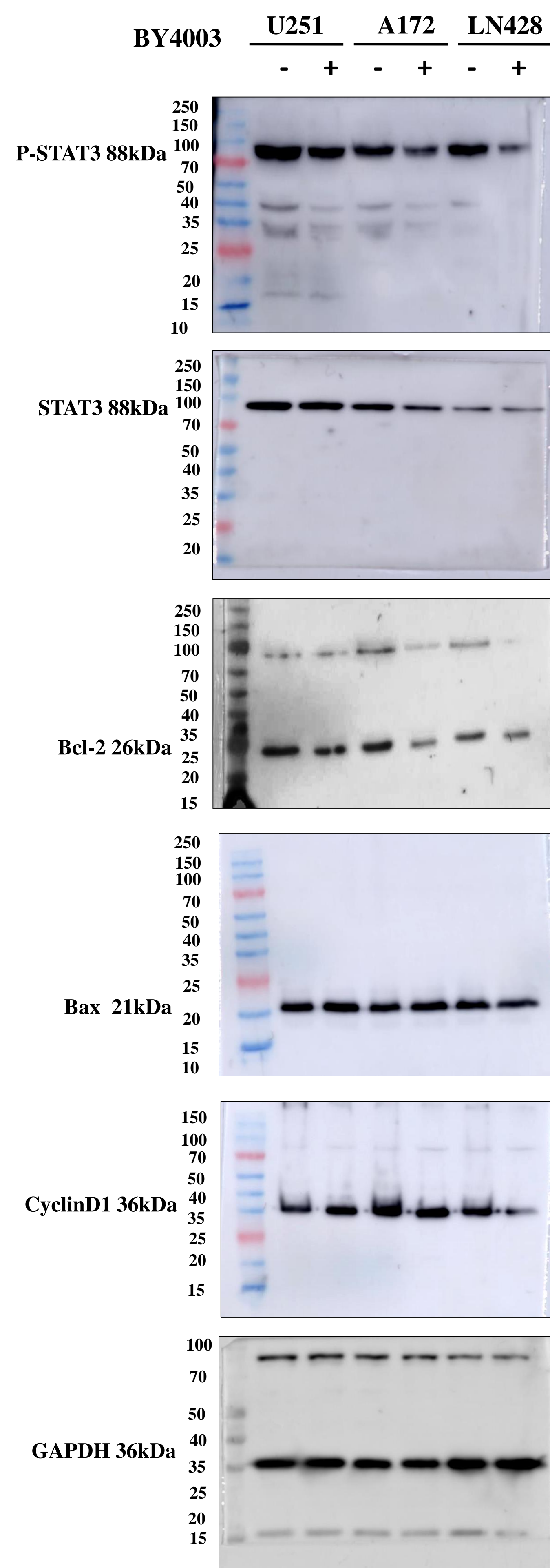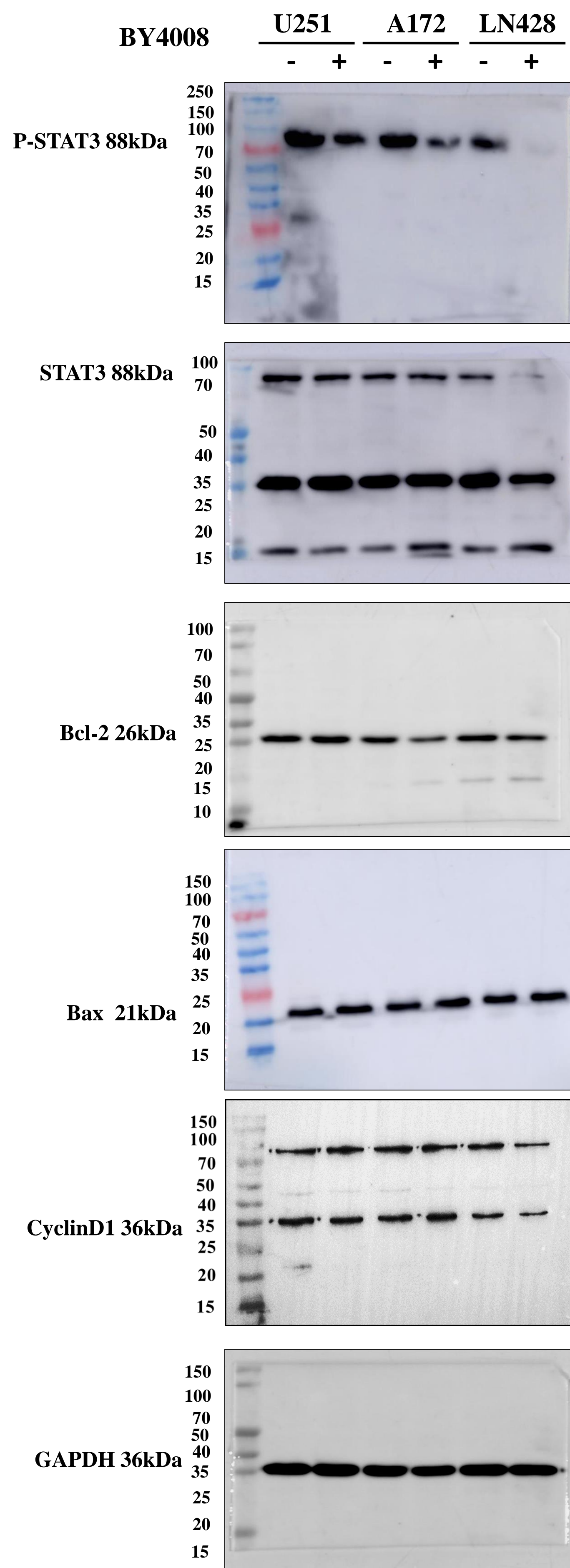

Supplement: Multimedia component 4 [file mmc4.pdf]
